# Sex-Specific Sub-Lethal Effects and Immune Response in *Ceratitis capitata* Wied. (Diptera: Tephritidae) Challenged with Spinosad

**DOI:** 10.3390/insects9030073

**Published:** 2018-06-21

**Authors:** Maria Elena Mura, Luca Ruiu

**Affiliations:** Dipartimento di Agraria, University of Sassari, 07100 Sassari, Italy; mariaelenamura@uniss.it

**Keywords:** spynosin, medfly, bioinsecticide, *Saccharopolyspora*, fecundity, bioassays, gene expression

## Abstract

The main objective of this study was to investigate the effects of the insecticidal compound spinosad on the survival, reproduction, and immune functions of the Mediterranean fruit fly. The lethal and sub-lethal effects were determined on *Ceratitis capitata* Wied. (Diptera: Tephritidae) challenged with different concentrations of spinosad. A median lethal concentration of 0.28 ppm was observed on flies feeding for 5 days on a treated diet. A significant and concentration-dependent decrease in fecundity, egg hatch rate, and lifespan was also detected in treated compared with control flies. Gene expression analyses conducted on treated insects by RT-qPCR revealed an immunomodulatory action of sub-lethal concentrations of spinosad. Target transcripts included several genes involved in medfly immunity and male or female reproductive functions. While a significant upregulation was detected in treated males a short time after spinosad ingestion, most target genes were downregulated in treated females. Our study confirmed the high toxicity of spinosad to *C. capitata*, highlighting an indirect effect on insect lifespan and reproductive performance at sub-lethal doses. In addition to defining the acute and sub-lethal toxicity of spinosad against the fly, this study provides new insights on the interaction of this compound with insect physiology.

## 1. Introduction

Before being commercialized in a specific country, all bioinsecticides based on insect pathogenic microorganisms and their metabolites undergo a strict approval procedure assessing their efficacy against target pests and ensuring an acceptable environmental impact.

Spinosad is a mixture of spinosyns A and D, tetracyclic polyketide aglycones including a neutral saccharide substituent, that are produced during the natural fermentation of the actinomycete *Saccharopolyspora spinosa* Mertz and Yao [1,2]. Given their potent insecticidal activity, and their safety for non-target organisms, spinosyns and their derivatives represent active substances successfully used for the integrated management of a wide range of target pests [3]. Whilst their mode of action is not completely understood, spinosyns were shown to interact with receptors of g-aminobutyric acid and nicotinic acetylcholine, activating a sequence of events leading to neuronal activity disruption, insect paralysis, and death [4]. When administered at sub-lethal concentrations, significant effects on survival, immature development, and reproduction have been observed on different insect species [5,6], which implies a direct or indirect interaction with insect physiology mechanisms. On the other hand, no information on the insect immune response activation as a consequence of spinosad challenges is available. Normally, insects respond to external challenges through the activation of their innate immune system [7]. This may involve either cellular (i.e., phagocytosis, encapsulation, nodulation) and humoral defense mechanisms depending on the type and intensity of the challenge. Among the latter, a significant role in stress tolerance is played by antimicrobial peptides (AMPs), reactive oxygen species (ROS), copper-containing enzyme phenol oxidase (PO), and heat shock proteins (e.g., HSP-70) [8,9,10]. Several studies have reported the immunomodulatory effects on insects challenged with diverse synthetic pesticides, including neonicotinoids, organochlorines, and organophosphates, whose main mode of action targets the nervous system [11].

Among the range of insect species showing susceptibility to spinosad, is the Mediterranean fruit fly, *Ceratitis capitata* Wied. (Diptera: Tephritidae). Significant efficacy of different formulations against the fly has been reported. Spinosad is successfully used for managing this fly [12,13]. This worldwide pest affects many fruits and vegetable crops, often achieving very high population densities and causing economically relevant damages to agricultural commodities [14]. Plant protection measures are therefore indispensable, and the development of integrated management strategies involving the use of environmentally responsible active substances is recommended [15].

While the insecticidal effects of spinosad are well documented, the effects of sub-lethal doses on the medfly immune system have not been investigated so far. The recent whole genome sequencing and annotation of *C. capitata* provided precious information on the genes possibly involved in defense mechanisms against insecticides and stress factors [16].

The main objective of this study was to investigate the immune reaction of medfly adults exposed to a spinosad-treated diet. For this purpose, the lethal and sub-lethal effects were preliminarily identified on both sexes. Successively, gene expression experiments were conducted to determine the main humoral response changes at the transcriptional level in challenged versus untreated insects.

## 2. Materials and Methods

### 2.1. Insect Bioassays

Laboratory bioassays were conducted employing newly emerged *C. capitata* adults provided by the rearing facilities of the Dipartimento di Agraria of the University of Sassari (Sassari, Italy), where a colony of this insect species is maintained using methods and conditions described elsewhere [17]. Experiments were conducted at 25 ± 1 °C with a photoperiod of L14:D10.

Spinosad technical mixture (CAS N. 168316-95-8, Sigma Aldrich, Saint Louis, MO, USA) was diluted in water to prepare fresh solutions of the required concentration for each bioassay.

A first experiment was conducted to assess the dose–response effects of spinosad. For this purpose, groups of 10 newly emerged adults, after being starved for 24 h, were maintained inside plastic cages (10 × 15 × 5 cm) and fed a 50 µL daily dose/cage of a sucrose solution (30%) containing spinosad. Food was administered through two capillary tubes (25 µL each) and control groups were fed with sucrose solution (30%). The experimental designed involved four replications with the following progressive range of spinosad concentrations: 1.2, 1.0, 0.8, 0.6, 0.4, 0.2, 0.1 ppm.

Mortality was assessed daily for 5 days and the whole experiment was repeated three times.

A second experiment was conducted to define the sub-lethal effects caused by spinosad by comparing fecundity, egg viability, and longevity in treated and untreated flies. Each experimental group (replicate) included 10 adults (five males and five females) maintained in transparent plastic boxes (10 × 15 × 5 cm) with a gauze covered window on one side that allowed females to lay eggs on a plate beneath containing water, from which eggs were collected for counting and hatching rate determination. This experiment was conducted with five replicates. Flies were fed by capillary tubes at the previously described daily doses using two sub-lethal spinosad concentrations (0.1 and 0.4 ppm) during the first 5 days, and just the sucrose solution afterwards. Control insects were fed the sucrose solution for the whole bioassay duration. Brewer’s hydrolyzed yeast (1 g) was added to each treated and untreated box as a source of protein. The number of eggs was counted from the sixth day on, for a 3-week period. Flies were checked daily to record mortality data. Egg hatching was determined by stereomicroscope observations on four replicated groups of 20 eggs from each insect cage 5, 10, and 15 days after first egg collection.

Pools of flies were submitted to genetic analysis after being exposed to a median lethal concentration of spinosad (0.3 ppm), according to the same experimental design of dose response bioassays. Flies from treated and control groups were sacrificed at progressive time intervals (6-12-24 h) from treatments. Three independent biological replicates (different fly samples) were involved in these experiments, and three technical replicates (replications within the same fly sample) were included in each analysis.

### 2.2. RT-qPCR Analysis

Insect pools were homogenized and total RNA was extracted employing TRIzol^®^ Reagent protocol (Life Technologies, Carlsbad, CA, USA) [18]. After being quantified by NanoDrop ND-1000 Spectrophotometer (Thermo Scientific, Waltham, MA, USA), RNA samples were treated with RQ1 RNase-Free DNase (Promega, Madison, WI, USA) and an aliquot (1 μg) of each was used for first-strand cDNA synthesis with oligo dT (Promega), SuperScript^®^ II Reverse Transcriptase (Life Technologies), and RNaseOUT™ Recombinant Ribonuclease Inhibitor (Life Technologies). Power SYBR^®^ Green PCR Master Mix (Life Technologies) was used in quantitative PCR reactions carried out on an Applied Biosystems 7900HT Fast Real-Time PCR System, with the following cycle conditions: denaturation at 95 °C for 10 min, 40 cycles of 95 °C for 15 s, annealing at 50–63 °C for 1 min, and extension at 60 °C for 1 min.

Forward and reverse primers were designed on target gene sequences deposited in GenBank (National Center for Biotechnology Information, NCBI) under the accession numbers provided in Table 1. Before being employed in qPCR analyses, each primer pair was tested by standard curve and dissociation curve analysis [19]. Tubulin and Glucose-6-phosphate dehydrogenase (G6PDH) were used as reference genes.

### 2.3. Data Analysis

Data were analyzed by using SAS software (version 9.1) with significance level set at α = 0.05 [20].

Mortality data were analyzed by Probit procedure to determine the median lethal concentration (LC50) [21].

Fecundity, longevity, and egg hatching data were subjected to one-way analysis of variance (ANOVA) followed by multiple comparison of means (adjust = Tukey).

The comparative 2^−ΔΔCt^ method [22] was used to analyze the relative expression of target genes. Data were normalized to internal control genes (tubulin and G6PDH) and comparatively analyzed using repeated measures ANOVA (PROC MIXED) with gene and time as factors, and means were separated using LSMEANS comparison (adjust = Tukey). Relative expression data of each gene were also analyzed by one-way ANOVA, followed by multiple comparison of means (adjust = Tukey).

## 3. Results

### 3.1. Lethal and Sub-Lethal Effects

**Figure 1:**
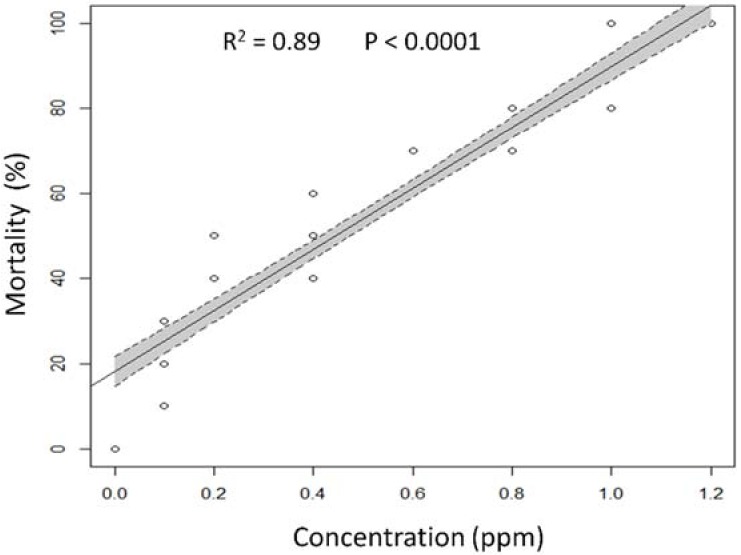
Linear regression plot with 95% confidence intervals (shaded areas) showing the predicted relationship between spinosad concentration and mortality of *Ceratitis capitata*.

The effects of spinosad on flies ingesting a treated diet were concentration dependent and, as shown in Figure 1, a positive correlation between concentration and acute toxicity was observed (adjusted *R*^2^ = 0.8904, F = 772.9, *p* < 0.0001). As a result of mortality percentages assessed on the fifth day, an LC_50_ (95% FL) of 0.28 (0.23–0.33) ppm (Slope ± SE = 1.90 ± 0.17; χ^2^ = 127.25; df = 1; *p* < 0.0001) was obtained.

When flies were challenged with sub-lethal concentrations of spinosad their fitness was significantly affected (Table 2). Flies exposed to spinosad showed reduced fecundity compared to the control, with halved values on flies treated with the higher concentration (F_2,12_ = 34.17, *p* < 0.0001). A significant decrease in the percentage of egg hatching was also observed in treated vs. control *C. capitata* (F_2,33_ = 22.88, *p* < 0.0001). The impact of treatments on insect longevity was significant, with a concentration dependent reduction of both male (F_2,12_ = 20.66, *p* = 0.0001) and female (F_2,12_ = 47.78, *p* < 0.0001) lifespan.

### 3.2. Gene Expression Analysis

**Figure 2:**
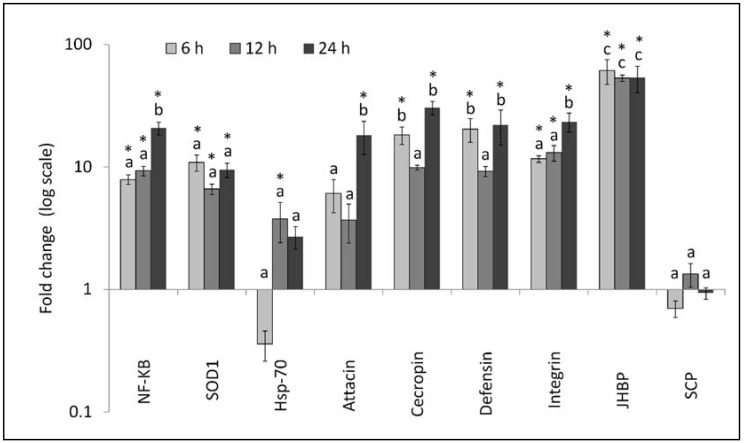
Relative expression profiles (mean ± SE) of immune-related genes of *Ceratitis capitata* males at different time intervals after spinosad treatments. Different letters above the bars indicate significantly different means (ANOVA (PROC MIXED), followed by LSMEANS comparison (adjust = Tukey), *p* < 0.001). Asterisks above bars indicate for each gene a significant difference with untreated control (ANOVA, Tukey adjusted *p* < 0.05).

The relative expression (fold change) of immune related genes in male flies exposed to spinosad for different time intervals (6, 12, and 24 h) are shown in Figure 2. Their expression level was significantly affected by the gene (F_8,72_ = 43.28, *p* < 0.0001) and time after spinosad challenge (F_2,144_ = 7.00, *p* = 0.0013). No significant gene × time interaction was observed (F_16,144_ = 1.16, *p* = 0.3106). A significant overexpression with respect to the untreated control was detected for *NF-kB* (F_3,32_ = 38.06, *p* < 0.0001), *SODCu/Zn* (F_3,32_ = 16.23, *p* < 0.0001), *Hsp-70* (F_3,32_ = 4.22, *p* = 0.0127), *attacin-B* (F_3,32_ = 6.16, *p* = 0.0020), *cecropin-1* (F_3,32_ = 25.49, *p* < 0.0001), *defensin-A* (F_3,32_ = 5.43, *p* = 0.0039), *integrin alpha-PS* (F_3,32_ = 16.25, *p* < 0.0001), and *JHBP_28C* (F_3,32_ = 6.77, *p* = 0.0012) genes. While for most genes upregulation was associated with all time detection intervals after treatments, in the case of *Hsp-70*, a slight though no- significant downregulation was detected 6 h after treatment. Furthermore, non-significant differences were observed between spinosad treatment and the control for *SCP_17a* gene (F_3,32_ = 1.97, *p* = 0.1386).

**Figure 3:**
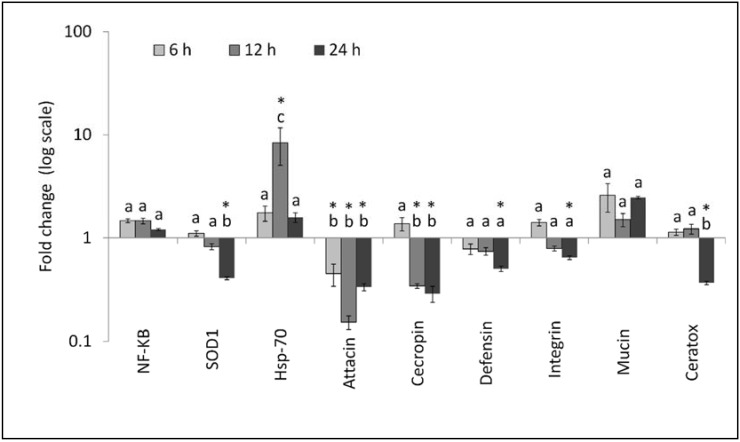
Relative expression profiles (mean ± SE) of immune-related genes of *Ceratitis capitata* females at different time intervals after spinosad treatments. Different letters above the bars indicate significantly different means (ANOVA (PROC MIXED), followed by LSMEANS comparison (adjust = Tukey), *p* < 0.001). Asterisks above bars indicate for each gene a significant difference with untreated control (ANOVA, Tukey adjusted *p* < 0.05).

The time-course gene expression in fly females is shown in Figure 3. The relative level of expression was significantly affected by the gene (F_8,72_ = 6.82, *p* < 0.0001) and time after treatment (F_2,144_ = 4.22, *p* = 0.0166). A significant gene × time interaction was also detected (F_16,144_ = 4.78, *p* < 0.0001). A significant upregulation was observed only for *Hsp-70*, which occurred 12 h after treatment (F_3,32_ = 4.39, *p* = 0.0108). Significant downregulation was observed 12 and/or 24 h post-treatment for *SODCu/Zn* (F_3,32_ = 19.96, *p* < 0.0001), *attacin-B* (F_3,32_ = 8.49, *p* = 0.0003), *cecropin-1* (F_3,32_ = 13.75, *p* < 0.0001), *defensin-A* (F_3,32_ = 7.89, *p* = 0.0004), *integrin alpha-PS* (F_3,32_ = 13.34, *p* < 0.0001), and *ceratotoxin A* (F_3,32_ = 17.06, *p* < 0.0001). There were non-significant changes for NF-kB (F_3,32_ = 1.13, *p* = 0.3507) and *mucin C* (F_3,32_ = 3.20, *p* = 0.0363).

## 4. Discussion

The susceptibility of *Ceratitis capitata* to spinosad is well documented [13,23]. A concentration-dependent effect was detected in our dose-response bioassays that showed lethal concentration values similar to those observed by other authors conducting experiments by ingestion [24]. The acute toxicity associated with just few ppm of the active substance supports the use of this bioinsecticide as an effective alternative to synthetic chemicals for the management of *C. capitata* [25,26]. When insects were exposed to sub-lethal concentrations, they exhibited reduced lifespan and fitness, with decreased fecundity and fertility rate. Consistent with our results, significant effects on adult survival and reproductive potential were observed on diverse insect species challenged with spinosad. Lopez et al. [5] detected a significant influence on the number of eggs per females of the bollworm *Helicoverpa zea* (Boddie) (Lepidoptera: Noctuidae) even though no correlation between treatment concentration and fecundity reduction was found. Our results are more in line with the outcome of other studies conducted on different species including the armyworm *Spodoptera exigua* (Hubner) (Lepidoptera: Noctuidae) [6], the diamondback moth *Plutella xylostella* (Lepidoptera: Yponomeutidae) [27], and the multicolored Asian lady beetle *Harmonia axyridis* (Coleoptera: Coccinellidae) [28], which highlighted a concentration dependent reduction of adult longevity, egg oviposition rate per female, and egg hatch ratio. Taken together, these outcomes corroborate the potent and broad-spectrum activity of spinosyns against diverse pest species, which relates to their unique though not completely understood mode of action targeting the insect nervous system [29]. On the other hand, surviving insects exposed to sub-lethal concentrations of spinosad exhibit reduced vigor, viability, and reproductive potential, which supports direct or indirect interactions with insect physiology. However, such mechanisms are not known and will need to be studied.

Dietary exposure of flies to spinosad at sub-lethal concentration affected the expression of genes involved in insect immunity mechanisms, with significant differences between males and females. Considerable immune-stimulatory effects in treated males were detected shortly after treatment, targeting the most common genes involved in the immune response of this species, like *attacin*, *cecropin*, *defensin*, and *integrin* [16,30]. Such overexpression also involved the genes encoding for protein products associated with the response to stress factors, including the heat shock protein Hsp-70 [31], the superoxide dismutase [Cu-Zn], an antioxidant enzyme involved in insect lifespan regulation [32], and the transcription factor NF-KB, which stimulates the expression of a wide range of functional genes [33]. On the other hand, downregulation of target antimicrobial peptides and other immune-related genes were observed soon after females were exposed to spinosad. The diverse immune response of different sexes might be related to a different level of susceptibility or simply to the acquisition of a different dose of spinosad due to a naturally higher rate of food intake in females [34].

In general, such immune response changes are consistent with the immunomodulatory effects derived from the sub-lethal action of a variety of natural and synthetic insecticidal substances, including spinosad [35]. For instance, in the case of the botanical compound azadirachtin, an antagonistic effect with the molting hormone was proposed [36], which may explain the downregulation of ovarian functions in treated flies [37,38]. Immunomodulatory activity was also detected in insects exposed to Pyriproxyfen and other insect growth regulators (IGRs) modulating the expression of immune related genes like prophenoloxidase and heat shock proteins [39,40]. Significant modifications in the cellular immune functions were observed as a consequence of treatments with the organochlorine endosulfan and the organophosphates dimethoate [41] and malathion [42]. Despite these studies, knowledge in this field is still limited and the mechanisms leading to the observed humoral immune defense alterations will have to be clarified [11].

Knowledge on spinosad mode of action is mostly focused on its interactions with insect nervous system [4]. However recent studies revealed an additional toxic action on non-neural insect cells, with induction of programmed cell death, autophagy, and mitochondrial dysfunctions [43,44].

Exposure to spinosad also determined a significant modulation of the expression level of genes related to *C. capitata* reproductive functions, with upregulation of JHBP_28C gene in treated males and downregulation of ceratoxin A gene in treated females compared with untreated controls. The modulation of these genes is likely to cause direct or indirect changes in the complex physiological mechanisms that regulate reproductive behavior [45]. JHBP proteins are carriers of the juvenile hormone (JH) released by corpora allata, so that an upregulation of their encoding genes in fly males will affect the JH level in the hemolymph, which is expected to cause mating behavior changes [46]. Ceratotoxins are cationic peptides secreted by *C. capitata* female reproductive accessory glands. The antibacterial properties associated with these peptides whose production is induced by the juvenile hormone stimulation suggest a protection role in the reproductive tract against bacterial infections [47]. The observed downregulation of *ceratotoxin A* gene in females challenged with spinosad corroborates the general immune impairment caused by this insecticidal compound on females exposed to sub-lethal doses.

In addition to a well-known acute insecticidal action, our results suggest that sub-lethal doses of spinosad may play a significant role in reducing *C. capitata* biotic potential through an indirect effect on its survival, development, and reproduction [27].

On the other hand, gene immunosuppression determined by spinosad may result in an increased susceptibility of insects to the action of abiotic and biotic factors, such as entomopathogenic microorganisms [48].

## 5. Conclusions

Our study confirmed the high toxicity of spinosad to *C. capitata*, highlighting an indirect effect on insect lifespan and reproductive performance at sub-lethal doses. In addition, the immunomodulatory changes observed in insects challenged with sub-lethal concentrations of spinosad provide new insights on the interaction of this compound with non-neural cells and the ability of its properties to affect insect physiology. Future studies will provide a deeper understanding of such interactions, thus contributing to maximize spinosad efficacy and safe usage in integrated pest management practices.

## Figures and Tables

**Table 1:** Primer pair sequences used to amplify *Ceratitis capitata* immune-related genes.

Protein Product	Gene ID	Accession Number ^a^	Primer Pair Sequences	Annealing Temperature (°C)
Heat shock protein	*Hsp-70*	Y08955.1	F: 5′CAAAGTGGAGATTATCGCCAACGACCA 3′ R: 5′CACTGTGATGACGGCGTCTGTTACTGT 3′	60
SODCu/Zn	*SOD1*	M76975.1	F: 5′CCCGTCCTAGTCACTGGTGAGGTTAAC 3′ R: 5′CTTCGTTGCTCCATCGCCACTGG 3′	62
Attacin-B	*attacin*	XM_004520300.2	F: 5′TGGAGCTCGCTAAAGGTGTGGGCACA3′ R: ′CTTTGTGAGCGAATCACTTATGCCGGGTGT 3′	63
Cecropin-1	*cecropin*	XM_004534275.2	F: 5′CTCCTCGCTGTCATCTTTGCCGTTTTC3′ R: 5′AGCAACATTGGCCGCCTGTTGGGC3	52
Defensin-A	*defensin*	XM_004537571.2	F: 5′TTATCGCCGGACTCATCCTCTGCTCCT3′ R: 5′ATGTACCGTTCCTGCAGTAACCGCCAC3′	63
Integrin alpha-PS	*integrin*	XM_004521659.1	F: 5′TGCAACCTGATCTGGGCGATTCGC3′ R: 5′ATGAGCTACTGCTATACGATGAGCCAGCCT3′	62
Mucin C	*mucin*	XM_004533242.1	F: 5′GGGGAGTACATAACCAATCCGGAAGCAACT3′ R: 5′GACTTGAGCGGTCGTTGTTGATGATGCAGT3′	60
Ceratotoxin A	*ceratotox*	AJ272446.1	F: 5′TAGTAGCCGAACCTGCTGCCGAAGATT3′ R: 5′AACGGGTAGAGCAGCCTTTGCAATGGGTAA3′	62
NF-kB	*NF-kB*	XM_004517947.2	F: 5′ACAAAGTTCTCAATGCCCACAATG3′ R: 5′GTTCCTTAACAGCGATATGTAGTGC3′	55
SCP_17a	*SCP_17a*	DQ406807.1	F: 5′AATATTCTTGTCGGCAACCATAA3′ R: 5′CAGAATTTGGCATGGATCAGT3′	50
JHBP_28C	*JHBP_28C*	DQ406809.1	F: 5′TGAACGGACCAGATATCCAA3′ R: 5′ACCATGCAGATAGGCAGGAC3′	52

^a^ GenBank accession number of sequences used for primer design.

**Table 2:** Means (±SE) of fecundity, egg hatching, and longevity of flies surviving exposure to sub-lethal concentrations of spinosad.

Spinosad Concentration (ppm) ^a^	Fecundity (No. Eggs/Female)	Egg Hatch ^b^ %	Longevity (Days) ^c^	
			Male	Female
Control	293.8 ± 17.1 ^a,d^	93.7 ± 1.1 ^a^	24.0 ± 1.0 ^a^	28.6 ± 0.8 ^a^
0.1	209.8 ± 11.8 ^b^	88.3 ± 0.9 ^b^	20.8 ± 0.9 ^b^	23.0 ± 0.8 ^b^
0.4	139.8 ± 9.3 ^c^	82.5 ± 1.4 ^c^	16.6 ± 0.5 ^c^	19.0 ± 0.7 ^c^

^a^ Spinosad was administered to flies only during the first 5 days of the experiment. From the sixth day on, flies were fed with just 30% sucrose solution, the same as the control. ^b^ Egg hatching was evaluated after 10, 15, and 20 days from adult emergence. Mean values are presented. ^c^ Days from adult emergence to adult death. ^d^ Means in each column followed by different letters are significantly different (ANOVA, Tukey test, *p* < 0.05).
